# Chinese herbal medicine for the treatment of intestinal cancer: preclinical studies and potential clinical applications

**DOI:** 10.1186/s12943-024-02135-3

**Published:** 2024-10-01

**Authors:** Juan Zhang, Yulin Wu, Yuanyang Tian, Hongxi Xu, Zhi-Xiu Lin, Yan-Fang Xian

**Affiliations:** 1 School of Chinese Medicine, Faculty of Medicine, The Chinese University of Hong Kong, Shatin, 999077 N.T., Hong Kong SAR China; 2 School of Pharmacy, Shanghai University of Traditional Chinese Medicine, Shanghai, 201203 P.R. China; 3 Hong Kong Institute of Integrative Medicine, The Chinese University of Hong Kong, Shatin, Hong Kong SAR China

**Keywords:** Chinese herbal medicine, Chinese herbal formulae, Anti-cancer effects, Clinical application, Intestinal cancer

## Abstract

Intestinal cancer (IC) poses a significant global health challenge that drives continuous efforts to explore effective treatment modalities. Conventional treatments for IC are effective, but are associated with several limitations and drawbacks. Chinese herbal medicine (CHM) plays an important role in the overall cancer prevention and therapeutic strategies. Recent years have seen a growing body of research focus on the potential of CHM in IC treatment, showing promising results in managing IC and mitigating the adverse effects of radiotherapy and chemotherapy. This review provides updated information from preclinical research and clinical observation on CHM’s role in treatment of IC, offering insights into its comprehensive management and guiding future prevention strategies and clinical practice.

**Supplementary Information:**

The online version contains supplementary material available at 10.1186/s12943-024-02135-3.

## Introduction

Intestinal cancer (IC), which includes colorectal cancer and small intestinal cancer, holds the third position among cancers globally and stands as the second leading cause of cancer-related deaths, and poses a significant public health concern [1–3]. Colorectal cancer encompasses cancers that occur in both the colon and the rectum, while colon cancer specifically refers to cancers that develop in the colon only, excluding those in the rectum. The risk factors for development of IC include the advanced age, genetic, environment, socioeconomic status, nutritional status, physical activity, and smoking [4]. Notably, there is a rising trend of younger individuals (< 50 years old) who are diagnosed with IC. Currently, the primary approaches for the treatment of IC involve laparoscopic surgical resection, complemented by radiotherapy and chemotherapy for advanced cases [5]. However, the prolonged use of these treatments often results in severe side effects, such as increasing resistance to chemotherapy, heightened metastasis, higher recurrence rate, and reduced quality of life of the patients, leading to treatment discontinuation. Additionally, chemotherapy commonly encounters challenges with treatment resistance [6]. In recent years, Chinese herbal medicine (CHM) has been getting increasing attention as a complementary therapy for digestive system tumors [7], owing to its several advantages, such as suppressing tumor progression, lowering treatment resistance, enhancing immune function, and mitigating the adverse effects of conventional therapies [8]. Different from the idea of Western medicine, Chinese medicine (CM) emphasizes a more holistic approach and the concept of “survival with tumor”, aiming not only to target the cancer cells and reduce the tumor size, but also to improve the quality of life of the cancer patients and extend the patient’s survival span [9].

### The characteristics and experience of CHM in the treatment of IC

CM, an ancient medical practice rooted in Chinese philosophy for over 2,000 years, is widely used for cancer treatment across Asia and some Western countries. CM aligns with the Chinese philosophy of Yin-Yang and Five Elements, emphasizing harmony of the human body with the external environment and advocating holistic well-being. Central to CM are the concepts such as five-zang and six-fu organs, qi (vital energy), blood, and meridians [10–12]. In CM diagnosis, the focus is on syndrome differentiation known as “Bian Zheng”, where a comprehensive analysis of observed clinical data enables formulating personalized treatment strategies. The main internal causes for condition such as IC in CM are typically attributed to Qi deficiency and exuberant toxic heat. Additionally, Qi deficiency often leads to dampness accumulation, resulting in evolving syndromes throughout the course of the disease. CM formulae are then frequently modified as signs and symptoms change along the course of the disease development. This approach is achieved by using ancient philosophies such as Yin-Yang, Five Elements and the roles of Jun (Monarch), Chen (Minister), Zuo (Assistant), and Shi (Messager) components of CM prescriptions [13]. The Jun herbs target primary symptoms, working synergistically with Chen herbs, and Zuo herbs mitigate adverse effects, while Shi herbs facilitate the delivery of the herbs to the target organs or harmonize all the herbs in the prescription to achieve optimal therapeutic action. The whole formula aims for a synergistic effect by directing active phytochemicals to their designated sites of action [14].

**Fig. 1:**
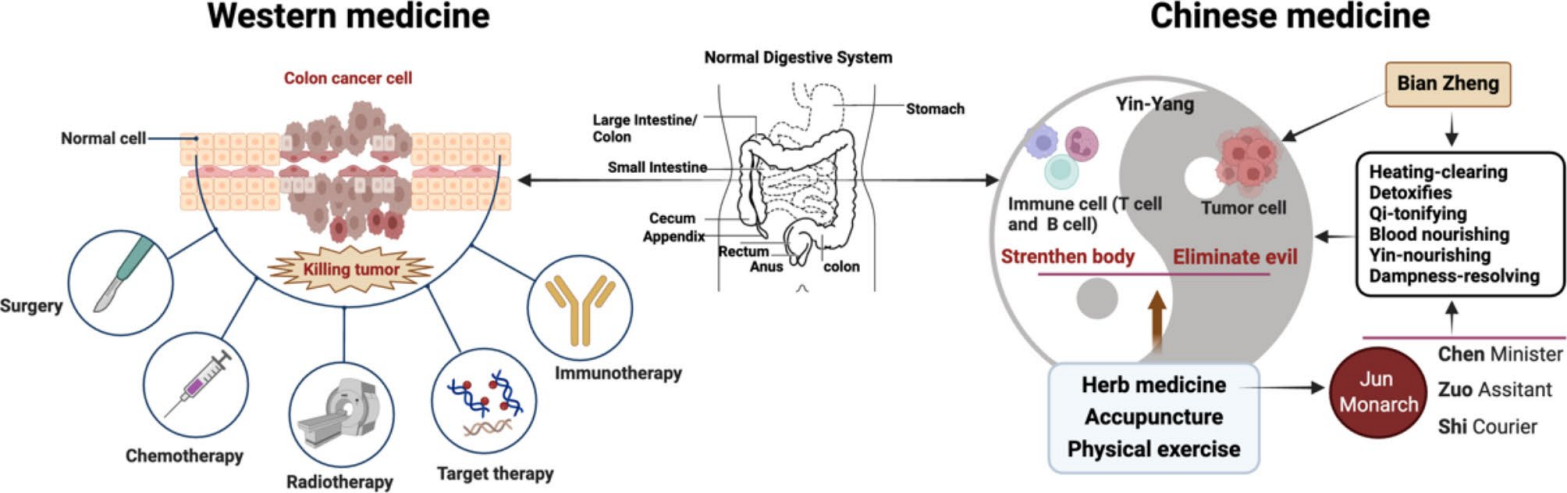
Comparison of the Western medicine and Chinese medicine treatment for IC

CM views cancer as a condition of Qi disturbance, treatable by regulating Qi, while Western medicine defines it as uncontrolled cell growth treated with surgery, chemotherapy, and radiotherapy (Fig. 1). While the efficacy of CHM is still debatable, an increasing amount of data indicates its potential in enhancing tumor response to chemotherapy and improving patient survival rates [10, 15]. Recent studies highlight CHM’s role in reducing IC incidence by disrupting cancer cell proliferation, inducing apoptosis, boosting immunity, and reducing treatment toxicities [16–18]. With the rising cancer rates and aging populations across the globe, exploring CHM’s benefits in cancer care is crucial. This review first summarized scientific literature on CHM’s preventive and therapeutic aspects on IC and its bioactive compounds. We then provided a brief overview of CHM products approved for clinical research. This review not only updates our understanding of the material basis of CHM’s anti-IC effect, but also serves as a resource for future empirical and clinical investigations.

### Preclinical study of CM single herbs and formulae against IC

**Fig. 2:**
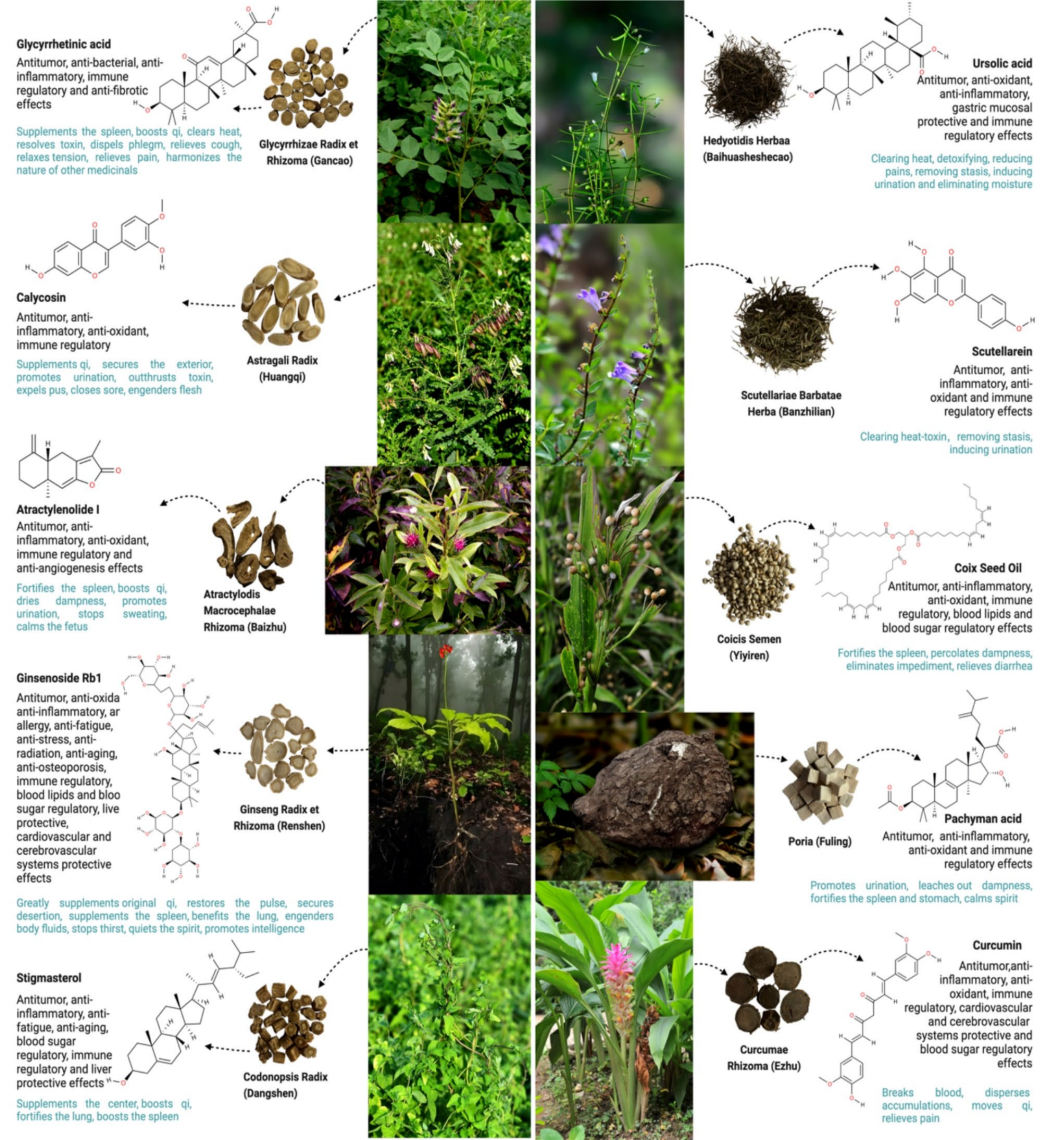
Elucidation of the potential therapeutic effects of the 10 most frequently utilized herbs and their functions. These herbal components could provide guidance for future drug discovery to combat IC

CM offers various treatment modalities, including herbal medicine, acupuncture & moxibustion, food therapy and physical exercise, with herb medicine being the most commonly applied. Unlike CM, Western medicine usually uses purified compounds to target a specific molecular pathway, while CM formulations often consist of multiple herbs and ingredients that aim for multiple targets and work through multiple pathways to achieve their therapeutic effects. To date, numerous herbs have been reported to be beneficial for patients with cancer, including IC [19]. In total, 61 classic Chinese medicine formulae (CMF) involve 119 single herbs and its represented 398 times (Table S1). Here we selected the top 10 herbs that are frequently incorporated in the 69 CMF that most commonly prescribed for the treatment of IC, and they are *Atractylodis Macrocephalae Rhizoma* (Baizhu in Chinese, 26, 46.62%), *Astragali Radix* (Huangqi in Chinese, 24, 349.34%), *Glycyrrhizae Radix et Rhizoma* (Gancao in Chinese, 23, 37.70%), *Poria* (Fuling in Chinese, 18, 29.51%), *Curcumas Rhizoma* (Ezhu in Chinese, 15, 24.59%), *Coicis Semen* (Yiyiren in Chinese, 13, 21.31%), *Hedyotidis Herba* (Baihuasheshecao in Chinese, 13, 21.31%), *Codonopsis Radix* (Dangshen in Chinese, 12, 16.97%), *Ginseng Radix et Rhizoma* (Renshen in Chinese, 5, 18.03%) and *Scutellariae Barbatae Herba* (Banzhilian in Chinese, 4, 14.75%) (Fig. 2 and Table S2). These herbs and CMF are known for their functions to strengthen body resistance, clear heat-toxin, eliminate dampness and resolve phlegm, and promote blood circulation to remove blood stasis.

Notably, herbs like *Glycyrrhizae Radix et Rhizoma*,* Astragali Radix*,* Atractylodis Macrocephalae Rhizoma*,* Ginseng Radix et Rhizoma*,* Codonopsis Radix* and *Curcumae Rhizoma* are frequently applied across various cancer via strengthen body resistance and promote blood circulation. *Hedyotidis Herba* and *Scutellariae Barbatae Herba* are commonly used in the early stages of IC (observational and mild stages) to clear the heat-toxin, while herbs such as *Coicis Semen* and *Poria*, which eliminate dampness and resolve phlegm, are commonly prescribed during the period of post-operative recovery. These herbal ingredients not only exert potential therapeutic benefits, but also serve as valuable references for future drug development targeting IC, as outlined in Fig. 2.

### CHM with the function of strengthening body resistance (SBR)

According to CM theories, the main pathogenesis of IC involves insufficiency of primordial Qi. Therefore, the most commonly used CHM for treating IC focuses on strengthening body resistance. This type of CHM can be beneficial throughout the entire stages of treatment by addressing general symptoms, boosting immunity, reducing chemotherapy-related side effects, enhancing chemotherapy tolerance, and lowering the risk of recurrence and metastasis. Among the top 10 frequently used CHM for IC, 5 are known for their immune-boosting property, including *Glycyrrhizae Radix et Rhizoma*, *Astragali Radix*, *Atractylodis Macrocephalae Rhizoma*, *Ginseng Radix et Rhizoma* and *Codonopsis Radix*. These herbs contain triterpene saponins, flavonoids and polysaccharides, and exhibit antitumor, anti-inflammatory, and immunoregulatory activities which are crucial for colorectal cancer (CRC) and colon cancer (CC) treatment.

*Glycyrrhizae Radix et Rhizoma* is renowned for its harmonizing property that improves herb synergy while providing Qi nourishment, pain relief, phlegm elimination and cough alleviation [20]. With over 20 triterpenoids and 300 flavonoids, it exhibits antiviral, antimicrobial, anti-inflammatory, and immunoregulatory properties, benefiting various systems such as neurological, gastrointestinal, respiratory, endocrine, and cardiovascular systems. Notably, compounds such as glycyrrhizin and glycyrrhetinic acid from *Glycyrrhizae Radix et Rhizome* have anti-inflammatory effect, and attenuate colorectal cancer pathogenesis [21–23]. *Astragali Radix* has the Qi-tonifying and vitality-boosting capabilities, and is frequently prescribed in CM practice for treating conditions including colds, diarrhea, and fatigue [24]. Its main components including calycosin, astragaloside IV, astragaloside III, and cycloastragenol, exerts diverse pharmacological benefits, particularly for immune, digestive, and respiratory systems. Studies have highlighted its antioxidant, immunomodulatory, and anti-inflammatory properties, with compounds such as astragaloside IV showing promise in reducing proinflammatory cytokines [25–27]. Calycosin, another chemical component of *Astragali Radix*, has demonstrated efficacy in inhibiting CRC cell proliferation, through ERβ-mediated regulation of the IGF-1R, PI3K/Akt signaling pathways [28]. Additionally, extract of *Atractylodis Macrocephalae Rhizoma* [29–32], *Ginseng Radix et Rhizoma* [33–43] and *Codonopsis Radix* [44, 45] were found to suppress the proliferation and induce ferroptosis and apoptosis in colorectal and colon cancer cells.

Si-Jun-Zi Decoction (Four Gentlemen Decoction), a well-known CMF, is usually used to treat various malignancies including IC, and may inhibit CRC liver metastasis by activating the innate immune system [46]. Additionally, Jianpi Huayu Decoction (Spleen-strengthening and Blood Stasis-Dissoving Decoction) has been shown to activate the p53-p21-Rb pathway, suppress cellular senescence in a CRC mouse model [47]. Moreover, Jianpi Jiedu Decoction significantly inhibited the cell proliferation and suppressed tumor cell migration, invasion, and angiogenesis by inhibiting the mTOR/HIF-1α/VEGF signaling pathway in CRC. It also improved the quality of life for CRC patients by reducing the adverse effects of conventional treatments, and improved outcomes when used alongside other therapies [48]. Additional details on the reported formulae for IC can be found in Table 1. These findings indicate the potential efficacy of the above single herbs and CMF in treating IC, and provide a rationale for their clinical use as promising therapeutic agents.

**Table 1:** Anti-IC effects and corresponding mechanisms of CMF

Functions	Clinical stage	CM formulae	Compounds/Constituents	Cancer type	Effect	Specific mechanisms	Ref.
SBR	Severe and critical stages	Si Jun Zi Decoction	*Ginseng Radix et Rhizoma* (Renshen), *Atractylodis Macrocephalae Rhizoma* (Baizhu), and *Poria* (Fuling)	CRC, CC	Inhibit the liver metastasis of CRC	Increase IFN-γ, IL-1α, IL-3 and GM-CSF; promotion of macrophage	[46, 81]
SBR	Mild and moderate stages	Jian Pi Hua Yu Decoction	*Pseudostellariae Radix* (Taizishen), *Coicis Semen* (Yiyiren), *Atractylodis Macrocephalae Rhizoma* (Baizhu), *Astragali Radix* (Huangqi), *Salviae Miltiorrhizae Radix et Rhizoma* (Danshen), *Scutellariae Barbatae Herba* (Banzhilian), *Paridis Rhizoma* (Chonglou), and *Curcumae Rhizoma* (Ezhu)	CRC	Inhibit the growth of CRC	Activate the p53-p21-Rb pathway	[47]
SBR	Mild and moderate stages	Jian Pi Jie Du Decoction	*Astragali Radix* (Huangqi), *Panacis Quinquefolii Radix* (Xiyangshen), *Atractylodis Macrocephalae Rhizoma* (Baizhu), *Poria* (Fuling), *Coicis Semen* (Yiyiren), *Smilacis Chinae Rhizoma* (Baqia), *Hedyotidis Herba* (Baihuasheshecao), *Scutellariae Barbatae Herba* (Banzhilian), *Paridis Rhizoma* (Chonglou), *Actinidia Argut* (Mihoutao), and *Glycyrrhizae Radix et Rhizoma* (Gancao)	CRC	Inhibit tumorigenesis, metastasis, and angiogenesis	Inhibit the mTOR/HIF-1α/VEGF signaling pathway.	[82]
SBR	Mild and moderate stage	Baizhu Huangqi Decoction	*Astragali Radix* (Huangqi), *Atractylodis Macrocephalae Rhizoma* (Baizhu), *Actinidia arguta* (Mihoutao), *Curcumae Radix* (Yujin), *Benincasae Exocarpium* (Dongguapi), and *Ficus Pumila* (Bili)	CRC	Inhibit migration and vasculogenic	Inhibit ROS/HIF-1α/MMP2 Pathway	[83]
SBR	Mild and moderate stages	Xiang Sha Liu Jun Zi Decoction	*Aucklandiae Radix* (Muxiang), *Amomi Fructus* (Sharen), Citri Reticulatae Pericarpium (Chenpi), *Pinelliae Rhizoma* (Banxia), *Ginseng Radix et Rhizoma* (Renshen), *Atractylodis Macrocephalae Rhizoma* (Baizhu), *Poria* (Fuling), *Glycyrrhizae Radix et Rhizoma* (Gancao), *Zingiberis Rhizoma* (Ganjiang), *Jujubae Fructus* (Dazao)	CRC, CC	Unknow	Unknow	[84, 85]
SBR	Mild and moderate stages	Shen Ling Bai Zhu San	*Lablab Aemen Album* (Baibiandou), *Ginseng Radix et Rhizoma* (Renshen), *Atractylodis Macrocephalae Rhizoma* (Baizhu), *Poria* (Fuling), *Glycyrrhizae Radix et Rhizoma* (Gancao), *Dioscoreae Rhizoma* (Shanyao), *Nelumbinis Semen* (Lianzi), *Platycodonis Radix* (Jiegeng), *Amomi Fructus* (Sharen), *Jujubae Fructus* (Dazao)	CRC, CC	Inhibit CRC cell growth	Repressing TGF-β-induced EMT program	[84, 86]
SBR	Mild and moderate stages	Yi Qi Fu Sheng Formula	*Codonopsis Radix* (Dangshen), *Atractylodis Macrocephalae Rhizoma* (Baizhu), *Poria* (Fuling), *Glycyrrhizae Radix et Rhizoma* (Gancao), *Myristicae Semen* (Roudoukou) and Akebia Fructus (Bayuezha)	CRC	Inhibits migration and invasion	Inhibiting the activation of ERK/MAPK signaling	[87]
SBR	Mild and moderate stages	Ba Zhen Decoction	*Ginseng Radix et Rhizoma* (Renshen), *Atractylodis Macrocephalae Rhizoma* (Baizhu), *Poria* (Fuling), *Angelicae Sinensis Radix* (Danggui), *Chuanxiong Rhizoma* (Chuanxiong), *Paeoniae Radix Alba* (Baishao), *Rehmanniae Radix Praeparata* (Shudihuang), and *Glycyrrhizae Radix et Rhizoma* (Gancao)	CRC	Enhancing the immune function against CRC	Promote T cell infiltration	[88]
SBR	Moderate, severe and critical stages	Si Ni Decoction	*Aconiti Lateralis Radix Praeprarata* (Fuzi), *Glycyrrhizae Radix et Rhizoma* (Gancao), and *Zingiberis Rhizoma* (Ganjiang)	CRC, CC	Suppressed CRC liver metastasis and alleviated liver injury	Upregulate IL-2, IFN-γ and downregulate IL-10 and TGF-β	[89, 90]
SBR	Moderate, severe and critical stages	Bu Shen Jian Pi Jie Du Decoction	*Astragali Radix* (Huangqi), *Rehmanniae Radix Praeparata* (Shudihuang), *Atractylodis Macrocephalae Rhizoma* (Baizhu), *Corni Fructus* (Shanzhuyu), *Dioscoreae Rhizoma* (Shanyao), *Sophorae Flavescentis Radix* (Kushen), *Vitis quinquangularis Rehd Folium* (Maoputaoye), and *Akebiae Caulis* (Mutong)	CRC	Enhance the efficacy of chemotherapeutical drugs oxaliplatin	Inhibit MAPK and the p-ERK/ERK pathway	[91]
SBR	Moderate, severe and critical stages	Fu Zheng Xiao Ai Decoction	*Astragali Radix* (Huangqi), *Atractylodis Macrocephalae Rhizoma* (Baizhu), *Poria* (Fuling), *Dioscoreae Rhizoma* (Shanyao), *Pseudostellariae Radix* (Taizishen), *Hedyotidis Herba* (Baihuasheshecao), *Pseudobulbus Cremastrae seu Pleiones* (Shancigu), *Chinese Actinidia Rhizoma* (Tengligen), *Glycyrrhizae Radix et Rhizoma* (Gancao)	CC	Ameliorate CRC cachexia	Activating Akt-mTOR pathway	[92]
SBR	Moderate, severe and critical stages	Dang Gui Bu Xue Decoction	*Astragali Radix* (Huangqi), *Angelicae Sinensis Radix* (Danggui)	CRC, CC	Inhibit metastasis CC progression	Upregulating Bax, Cas3, C-cas3, and downregulate Bcl2	[93]
CHT	Moderate, severe and critical stages	Pien Tze Huang	*Bovis Calculus* (Niuhuang), *Snake Gall* (Shedan), and *Notoginseng Radix et Rhizoma* (Sanqi)	CRC	Suppress CRC carcinogenesis	Improve gut barrier function	[57]
CHT	Mild, moderate, severe and critical stages	Wei Tong Xin	*Rhei Radix et Rhizoma* (Dahuang), *Pharbitidis Semen* (Qianniuzi), *Auchlandiae Radix* (Muxiang), Gleditsia sinensis Lam. (Zaojia) and *Glycyrrhizae Radix et Rhizoma* (Gancao)	CRC	Inhibit tumor growth	Inhibit PI3K/AKT signaling	[94]
CHT	Moderate, severe and critical stages	Ban Xia Xie Xin Decoction	*Pinelliae Rhizoma* (Banxia), *Scutellariae Radix* (Huangqin), *Zingiberis Rhizoma* (Ganjiang), *Ginseng Radix et Rhizoma* (Renshen), *Glycyrrhizae Radix et Rhizoma* (Gancao), *Coptidis Rhizoma* (Huanglian), *Jujubae Fructus* (Dazao)	CRC, CC	Inhibits proliferation and tumor growth	Inhibition of the PI3K/AKT/mTOR axis	[95, 96]
CHT	Moderate, severe and critical stages	Xian Lian Jie Du Decoction	*Agrimoniae Herba* (Xianhecao), *Coptidis Rhizoma* (Huanglian), *Sophorae Flavescentis Radix* (Kushen), *Coicis Semen* (Yiyiren), *Sparganii Rhizoma* (Sanleng), *Curcumae Rhizoma* (Ezhu), *Astragali Radix* (Huangqi) and *Atractylodis Macrocephalae Rhizoma* (Baizhu)	CRC, CC	Inhibit the occurrence of CC inner wall tumors	Reduce the inflammation level, alleviating intestinal microbial flora imbalance and metabolic disorders	[97, 98]
CHT	Mild, moderate, severe and critical stages	Huang Lian Jie Du Decoction	*Coptidis Rhizoma* (Huanglian), *Phellodendri Amurensis Cortex* (Guanhuangbo), *Gardeniae Fructus* (Zhizi), *Scutellariae Radix* (Huangqin)	CRC	Improve 5-Fu induced diarrhea and tumor inhibition	Activating Wnt/β-catenin signaling	[99]
CHT	Moderate, severe and critical stages	Jie Du San Gen Decoction	*Ginseng Radix et Rhizoma* (Renshen), *Atractylodis Macrocephalae Rhizoma* (Baizhu), *Poria* (Fuling), *Glycyrrhizae Radix et Rhizoma* (Gancao)	CRC, CC	Reverse 5-FU resistance	Activate PI3K/AKT/HIF-1*α* signaling pathway	[100, 101]
CHT	Mild, moderate, severe stages	Xiao Ai Jie Du Decoction	*Scutellariae Barbatae Herba* (Banzhilian), *Pseudostellariae Radix* (Taizishen), *Ophiopogonis Radix* (Maidong), *Cremastrae Pseudobulbus Pleiones Pseudobulbus* (Shancigu), *Curcumae Rhizoma* (Ezhu), *Akebiae Fructus* (Yuzhizi)	CC	Unknow	Unknow	[102]
CHT	Mild, moderate, severe stages	Huang Qin Decoction	*Scutellariae Radix* (Huangqin), *Paeoniae Radix Alba* (Baishao), *Jujubae Fructus* (Dazao), *Glycyrrhizae Radix et Rhizoma* (Gancao)	CRC	Attenuate colitis, reduce tumor burden	Suppress PI3K/Akt pathway	[59]
CHT	Mild, moderate, severe stages	San Wu Huang Qin Decoction	*Scutellariae Radix* (Huangqin), *Sophorae Flavescentis Radix* (Kushen), *Rehmanniae Radix Praeparata* (Shudihuang)	CRC	Inhibit CRC tumorigenesis	Suppress tlr4/NF-κb	[60]
CHT	Moderate, severe and critical stages	Ge Gen Qin Lian Decoction	*Puerariae Lobatae Radix* (Gegen), *Scutellariae Radix* (Huangqin), *Coptidis Rhizoma* (Huanglian), *Glycyrrhizae Radix et Rhizoma* (Gancao)	CRC	Enhance anti- tumor immunity	Increase CD8 + T cells and the expression of IFN-γ	[103]
CHT	Mild, moderate and severe stages	Da Cheng Qi Decoction	*Rhei Radix et Rhizoma* (Dahuang), *Magnoliae Officibalis Cortex* (Houpo), *Aurantii Fructus Immaturus* (Zhishi), *Natrii Sulfas* (Mangxiao)	CRC	Unknow	Unknow	[104]
CHT	Mild and moderate stages	Teng Long Bu Zhong Decoction	*Chinese Actinidia* (Tengligen), *Solani Nigri Herba* (Longkui), *Duchesnea indica* (Shemei), *Atractylodis Macrocephalae Rhizoma* (Baizhu), *Poria* (Fuling), *Coicis Semen* (Yiyiren), *Visci Herba* (Hujisheng), *Scutellariae Barbatae Herba* (Banzhilian)	CC	Induce CRC senescence	Upregulation of p21(WAF1/CIP1) and p16	[105]
CHT	Mild and moderate stages	Zuo Jin Wan	*Coptidis Rhizoma* (Huanglian) and *Euodiae Fructus* (Wuzhuyu)	CRC	Inhibit proliferation and induce apoptosis	Inhibit PI3K-Akt signaling pathway	[106]
EDRP	Moderate and severe stages	Er Chen Decoction	*Pinelliae Rhizoma* (Banxia), *Citri Reticulatae Pericarpium* (Chenpi), *Poria* (Fuling), *Glycyrrhizae Radix et Rhizoma* (Gancao)	CRC	Inhibit proliferation and induce apoptosis	Inhibit MAPK-STAT signaling	[65]
PBCRBS	Mild and moderate stages	Shao Yao Decoction	*Paeoniae Radix Alba* (Baishao), *Angelicae Sinensis Radix* (Danggui), *Coptidis Rhizoma* (Huanglian), *Arecae Semen* (Binlang), *Aucklandiae Radix* (Muxiang), *Glycyrrhizae Radix et Rhizoma* (Gancao), *Rhei Radix et Rhizoma* (Dahuang), *Scutellariae Radix* (Huangqin), *Cinnamomi Cortex* (Rougui)	CRC	Inhibit proliferation and induce apoptosis	Suppress inflammation and EMT	[68]
PBCRBS	Mild and moderate stages	San Jie Yi Liu Formula	*Pinelliae Rhizoma Preparatum* (Fabanxia), *Sarcandrae herba* (Zhongjiefeng), *Fritillariae Thunbergii Bulbus* (Zhebeimu), *Eupolyphage Steleophaga* (Tubiechong)	CRC	Inhibit proliferation and induce apoptosis	Downregulate cyclin D1, CDK4 and BCL-2	[69]

SBR: strengthening body resistance; CHT: clearing heat-toxin; EDRP: eliminating dampness and resolving phlegm; PBCRBS: promoting blood circulation for removing blood stasis; HMGB1: high-mobility group box 1.↓: Decreasing or down-regulation ↑: Increasing, activating or up-regulation.

### CHM with the functions of clearing heat and toxin (CHT)

Another pathogenesis of IC is brewing amassment of heat toxin. The main treatment principle should be “clearing heat-toxin”. Simultaneously, the treatment strategy emphasizes strengthening the body’s resistance and detoxifying without damaging the Qi. However, herbs that clear heat-toxin typically have a cold and bitter taste. Pattern identification as the basis for determining treatment strategies should be strictly followed for patients with the pattern of spleen-stomach vacuity cold. Therefore, these herbs are often used in the early stages of IC because the Qi has not been severely affected at this stage. Among the top 10 frequently used CHM for IC, *Hedyotidis Herbaa* and *Scutellariae Barbatae Herba* are often prescribed.

*Hedyotidis Herba*, a well-known CHM with heat-clearing, detoxification and promoting blood circulation properties, is employed in treating inflammation-related diseases, such as hepatitis, appendicitis, urethritis and malignant tumors such as CRC [49, 50]. Studies have shown that its ethanolic extract can suppress the metastasis of 5-Fluorouracil (5-FU)-resistance CRC cells by regulating TGF-β/SMAD4 signaling pathway [51]. Additionally, this extract induces apoptosis through the mitochondrion-dependent pathway in human colon carcinoma cells [52]. Its major component, ursolic acid exhibits significant anti-tumor activity in COLO 205 colon cancer cells [53]. *Scutellariae Barbatae Herba* plays a crucial role in numerous medicinal formulae utilized for treating various cancers [54]. Scutellarein, isolated from *Scutellariae Barbatae Herba*, demonstrated to trigger apoptosis in colon cancer HCT 116 cells by increasing intracellular ROS production, leading to mitochondrial membrane collapse [55]. Furthermore, polysaccharides of *Scutellariae Barbatae Herba* can hinder the proliferation, induce apoptosis and impede the EMT process in human colon cancer HT29 cells through inhibition of PI3K/AKT signaling pathway [56].

Pien Tze Huang (PZH) is a famous Chinese patented herbal medicine, and has been shown to suppress CRC carcinogenesis in a dose-dependent manner in mice [57]. Transcriptomic analysis revealed that PZH inhibited PI3K-Akt, interleukin-17, tumor necrosis factor, and cytokine-chemokine signaling by manipulating gut microbiota and metabolites, improving gut barrier function, and suppressing oncogenic and pro-inflammatory pathways, thereby inhibiting CRC carcinogenesis [57]. Jie Du San Gen Decoction could reverse 5-FU resistance by suppressing glycolysis through the PI3K/AKT/HIF-1*α* signaling pathway, inducing apoptosis and enhancing anti-tumor activity [58]. Huang Qin Decoction has been shown to ameliorate colitis, lower tumor burden, and promote cell apoptosis in CRC mice by suppressing PI3K/Akt pathway, and inhibit the proliferation, migration, and invasion of CRC cells [59]. San Wu Huang Qin Decoction effectively inhibited tumorigenesis and protected the mucosal barrier in CRC, partially by targeting gut microbiota, and the findings support its clinical use for the prevention and treatment of IC [60]. Detailed information about the most common CMF used for IC treatment is summarized in Table 1.

### CHM with the functions of eliminating dampness and resolving phlegm (EDRP)

In CM, the concept of “phlegm turbidity” is closely associated with the metastasis of IC. Consequently, it is common to combine herbal medicines that eliminate dampness and resolve phlegm at various stages of IC. This approach aims to strengthen the regulation of Qi, resolve phlegm, promote blood circulation to remove blood stasis for treating cremation phlegm. Two commonly used CHM with dampness-eliminating and phlegm-resolving properties in IC treatment are *Coicis Semen* and *Poria*.

*Coicis Semen* could strengthen the spleen functions and eliminate the accumulation of dampness heat in the body. It has often been used to treat diseases such as dysuria, edema, spleen deficiency-related diarrhea, rheumatism, neuralgia, and acute abdominal inflammation [61]. Kanglaite, isolated from *Coicis Semen*, has been reported to inhibit EMT caused by TNF- via inhibiting the activation of NF- in colorectal cancer cells [62]. *Poria* has historical use in treating edema, sputum, palpitation, and insomnia [63]. Its main components, polysaccharides, include carboxymethylated pachyman, could modulate the intestinal flora balance and reduce colon damage induced by 5-FU in CT26 tumor-bearing mice. The mechanisms were believed to involve the regulation of NF-B, Nrf2-ARE, and MAPK/P38 pathways [64].

Er Chen Decoction (Two Olds Decoction), a well-known CMF, exerts metabolism-regulating, immunoregulatory, and anti-tumor properties, along with the ability to eliminate dampness and resolve phlegm. Studies have shown that Er Chen Decoction could inhibit CRC cell proliferation by blocking cell cycle and promoting cell apoptosis, and suppress the tumor growth in mice by inhibiting MAPK-STAT signaling pathway [65].

### CHM with the function of promoting blood circulation to remove blood stasis (PBCRBS)

A prevalent syndrome observed in CHM among IC patients is the dual vacuity of the spleen and kidney. Due to the prolonged tumor course, patients often present with blood deficiency, leading to a complex interplay of deficiency and excess. Hence, it is feasible to select CHM with the functions of invigorating the spleen and replenishing Qi, promoting blood circulation to remove blood stasis to treat both manifestation and root cause of the disease.

*Curcumae Rhizoma* is the most frequently used herb in the CMF clinically employed to treat tumors and fibrosis. It has diverse pharmacological effects, including anti-tumor, antithrombosis, regulation of blood lipid, lowering blood glucose and antioxidant properties [66]. Its primary constituents, including volatile oil and curcumin, have shown inhibitory effects on colon cancer growth both in in vivo and in vitro models. They were also found to contribute to reducing tumor angiogenesis, improved tumor vessel structures and normalized tumor vessels [67]. Notable formulae incorporating *Curcumae Rhizoma* include Shao Yao Decoction and Sanjie Yiliu Formula.

Shao Yao Decoction, a traditional CMF, is known for its effectiveness in treating ulcerative colitis. Recent studies have shown that it significantly increased the survival rate of mice, improved their overall health and reduced the incidence and number of colonic neoplasms by inhibiting epithelial–mesenchymal transition (EMT) signaling transduction and attenuating pro-inflammatory cytokines [68]. On the other hand, San Jie Yi Liu Formula selectively decreased the viability of CRC cell lines without affecting normal human kidney cells, and significantly suppressed proliferation and induced apoptosis by downregulating cyclin D1 and CDK4, while upregulating BCL-2 expression [69].

**Fig. 3:**
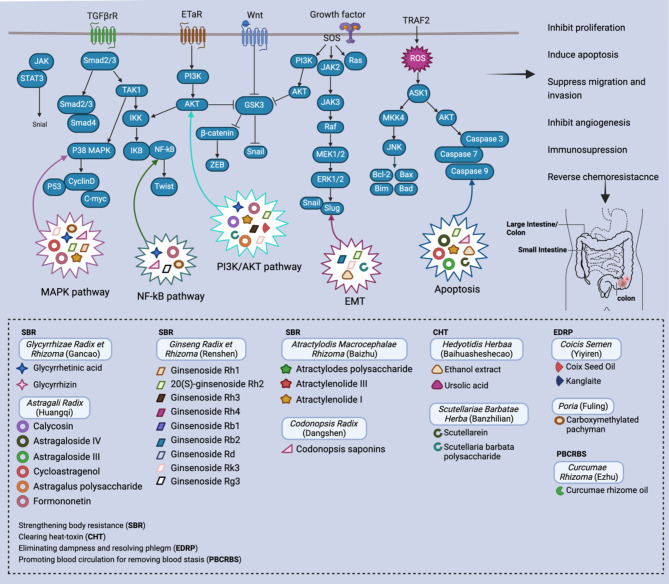
Schematic presentation of the effects and molecular mechanisms of herbs and their ingredients in treating IC. This includes the regulation of cancer cell proliferation, apoptosis, adhesion and migration, inhibition of tumor angiogenesis and reverse of chemoresistance. The active constituents of these herbs affect numerous molecular processes, influence key intracellular signaling regulators such as MAPK, NF-κB, PI3K/AKT and EMT, all of which are crucial in the development and progression of IC

In summary, herbs, their ingredients and CMF that utilize SBR, CHT, EDRP and PBCRBS have demonstrated great potential in the treatment of IC. Mechanistically, as above alluded to, the anti-IC activities are related to the inhibition of the proliferation, migration and, angiogenesis, induction of apoptosis, and the modulation of the host immune system. The corresponding molecular mechanisms for these effects are summarized in Fig. 3 and Table 2. Detailed information on the anti-IC effects of CMF is summarized in Table 1.

**Table 2:** Effects and specific mechanisms of single herbs and their ingredients for the treatment of IC

Functions	Clinical stage	CHM	Ingredients	Cancer	Model	Effect	Specific mechanism	Ref.
SBR	Observational, mild, moderate, severe and critical stages	*Glycyrrhizae Radix et Rhizoma* (Guanaco in Chinese)	Glycyrrhetinic acid	CRC	In vitro (LoVo, SW480, SW620)/ In vivo (BALB/c)	Inhibit proliferation and migration	↓PI3K and STAT3 signaling pathway	[70]
				CRC	In vitro (HT29, Caco-2, SW480)/ In vivo (BALB/c)	Enhance immunity	↓GSH-dependent GPX4 expression	[71]
			Glycyrrhizin	CC	ICR mice	Inhibit proliferation and dedifferentiation	↓HMGB1-TLR4-NF-κB signaling	[23]
SBR	Observational, mild, moderate, severe and critical stages	*Astragali Radix* (Huangqi in Chinese)	Calycosin	CRC	In vitro (SW480, LoVo) In vivo (nude mice)	Induce apoptosis	↓IGF-1R and PI3K/AKT signaling	[72]
			Astragaloside IV	CRC	In vitro (SW480, HT29, NCM460, SW620, CT26, HCT116) In vivo (BALB/c)	Suppress CRC growth	↓Cyclin D1, CDK4, Bcl 2, B7-H3	[73]
			Astragaloside III	CRC	In vitro (CT26, NK cells) In vivo (BALB/c)	Impede tumor growth	↑ IFN-γ secretion of NK cells	[74]
			Cycloastragenol	CC	In vitro (HT29, HCT116)	Inhibit proliferation	↑p53 activation	[75]
			Astragalus polysaccharide	CC	In vitro (4T1, CT26) In vivo (BALB/c)	Overcome tumor immune tolerance	↓PD-L1, AKT/mTOR/p70S6K	[76]
			Formononetin	CRC	In vitro (RKO, SW1116, HCT116) In vivo (BALB/c)	Suppress angiogenesis	Decrease the expression of VEGF	[77]
SBR	Observational, mild, moderate, severe and critical stages	*Atractylodis Macrocephalae Rhizoma* (Baizhu in Chinese)	Atractylodes polysaccharide	CRC	In vitro (MC38, CT26) In vivo (TLR4 KO C57BL/6)	Tumor suppression and immune regulation	↓MyD88/TLR4 signaling	[29]
			Atractylenolide I	CRC	In vitro (HCT116, SW480) In vivo (BALB/c)	Induce apoptosis	↓JAK2/STAT3 signaling	[31]
				CC	In vitro (HT29)	Inhibit proliferation and induce apoptosis	↓Bcl-2 ↑Bax, Bak, Bad, Bim, Bid, Puma, cleaved caspase 9, cleaved caspase 3, cleaved caspase 7 and cleaved PARP	[32]
			Atractylenolide III	CRC	In vitro (HCT116) In vivo (BALB/c)	Induce apoptosis	↓Bcl-2 ↑Bax, cleaved caspase-3 and p53	[30]
SBR	Observational, mild, moderate, severe and critical stages	*Ginseng Radix et Rhizoma* (Renshen in Chinese)	Ginsenoside Rh3	CRC	In vitro (HT29, HCT116, SW620, DLD1, RKO, HCoEpiC) In vivo (BALB/c)	Induces pyroptosis and ferroptosis	↓Stat3/p53/NRF2	[33]
			Ginsenoside Rb2	CRC	In vitro (HCT116, SW620) In vivo (nude mice)	Inhibit growth and metastasis	↓TGF-β1/Smad signaling	[34]
			Ginsenoside Rh4	CRC	In vitro (HT29, HCT116, DLD1, RKO) In vivo (nude mice)	Inhibit proliferation and induce apoptosis	↑ ROS/p53 signaling	[35]
			Ginsenoside Rg3	CRC	In vivo (nude mice)	Suppressing angiogenesis	↓NF-κB (VEGF, CD31, COX-2)	[36]
				CC	In vitro (SW48, HCT15) In vivo (nude mice)	Inhibit metastasis	↓Notch-Hes1-EMT signaling	[37]
			Ginsenoside Rd	CRC	In vitro (HT29, SW620) In vivo (NSG mice (NOD.Cg-Prkdc^scid^ Il2rg^tm1Wjl/SzJ^, The Jackson Laboratory)	Inhibit metastasis	↓EGFR signaling	[38]
			20(S)-ginsenoside Rh2	CRC	In vitro (HCT15, HCT116, DLD1, CCD-18Co) In vivo (BALB/c)	Inhibit tumor growth	↓Axl signaling	[39]
				CC	In vitro (HCT116, SW620, SW480, CaCo-2) In vivo (BALB/c)	Inhibit tumor growth	↓miR-150-3p/SRCIN1/Wnt axis	[40]
			Ginsenoside Rh1	CRC	In vitro (SW620) In vivo (BALB/c)	Inhibit invasion and migration	↓MAPK signaling	[41]
			Ginsenoside Rk3	CRC	In vivo (C57BL/6)	Immune regulation	↓JAK-STAT3 signaling	[42]
			Ginsenoside Rb1	CC	In vivo (BALB/c)	Reducing inflammation	↓TNF-α, IL-6	[43]
SBR	Observational, mild, moderate, severe and critical stages	*Codonopsis Radix* (Dangshen in Chinese)	Codonopsis saponins	CC	In vitro (HCT116, SW480) In vivo (BALB/c)	Induce apoptosis	↑ NF‑κB signaling	[44]
				CC	In vitro (HT-29)	Induce cell cycle arrest and apoptosis	↑ ROS generation	[45]
CHT	Observational and mild stages	*Hedyotidis Herbaa* (Baihuasheshecao in Chinese)	Ethanol extract	CRC	In vitro (HCT-8)	Inhibit metastasis	↓TGF‑β (SMAD4, N‑cadherin, E‑cadherin)	[78]
				CRC		Inhibit proliferation and metastasis	↓PI3K/AKT signaling	[79]
				CC	In vitro (HT29)	Induce apoptosis	Activation of the mitochondrion-dependent pathway	[52]
CHT	Observational and mild stages	*Scutellariae Barbatae Herba* (Banzhilian in Chinese)	Scutellarein	CC	In vitro (HCT116)	Induce apoptosis	↑ ROS, caspase-3, Bcl-2	[55]
			Scutellaria barbata polysaccharide	CC	In vitro (HT29)	Inhibit proliferation and metastasis	↓EMT, PI3K/AKT	[56]
EDRP	Recovery stage	*Coicis Semen* (Yiyiren in Chinese)	Coix Seed Oil	CRC, CC	In vitro (HT29, Caco-2, HCT116)	Induce cell cycle arrest and apoptosis	↓PI3K/AKT signaling	[80]
EDRP	Recovery stage	*Poria* (Fuling in Chinese)	Carboxymethylated pachyman	CRC	In vivo (CT26 tumor-bearing mice)	Reduce intestinal mucositis	↓NF-κB, Nrf2-ARE and MAPK/P38	[64]
PBCRBS	Observational, mild, moderate, severe and critical stages	*Curcumae Rhizoma* (Ezhu in Chinese)	Curcumae rhizome oil	CC	In vitro (HUVECs, HCT116) In vivo (BALB/c nude mice)	Inhibit tumor growth and angiogenesis	↓VEGFA (VE-cadherin, CD31)	[67]

CRC: Colorectal cancer; GM-CSF: Granulocyte-macrophage colony-stimulating factor.

### Progress in clinical trials of CHM on IC

The current progress of CM application in clinical studies for treating IC demonstrates promising results. Despite the success observed in cell culture and preclinical studies with CHMs, the goal of the basic research is to apply these agents in clinical setting. Randomized controlled trials (RCTs) are crucial for validating the efficacy of CHMs in IC treatment [107], and ongoing trials are revealing the therapeutic potential of various CHM formulations and isolated chemical compounds (Table 3). These trials have provided important insights into the anti-cancer properties of CHM ingredients, instilling confidence in their potential therapeutic benefits against IC. The progress in clinical studies contributes to the broader efforts of modernization of CHM, facilitates its integration into global cancer care, accelerates the clinical translation of CHM products for IC treatment, and offers new possibilities for personalized and evidence-based cancer care.

**Table 3:** Clinical studies of CMF for IC treatment

CHM/Herbs	Study title	Study design	Cancer	Phase	Status	Sponsor	Ref/NCT no.
Shenbai Granules	A Randomized Clinical Trial of Shenbai Granules in Reducing Recurrence of Colorectal Adenoma	400 participants, Randomized, Parallel Assignment	Colorectal	II-III	Completed	Jiangsu Famous Medical Technology Co., Ltd.	NCT03616444
Jianpi Huatan dispensing granule	Effect of Jianpi Huatan Decoction on Advanced Colorectal Cancer	200 participants, Randomized, Parallel Assignment	Colorectal	III	Recruiting	Xiyuan Hospital of China Academy of Chinese Medical Sciences	NCT05187481
XLJDOD compound granule	Xian-Lian-Jie-Du Optimization Decoction as an Adjuvant Treatment for Prevention of Recurrence of Colon Cancer	730 participants, Randomized, Parallel Assignment	Colon	III	Recruiting	Jiangsu Famous Medical Technology Co., Ltd.	NCT05709249
Huaier Granule	Huaier Granule as Adjuvant Therapy for Colorectal Cancer After Radical Surgery	230 participants, Randomized, Parallel Assignment	Colorectal	III	N/R	Sixth Affiliated Hospital, Sun Yat-sen University	NCT02796820
Jianpi Huatan dispensing granule	Strengthening the Spleen and Reducing Phlegm Method in Improving Radical Resection Rate of Colorectal Cancer	350 participants, Randomized, Parallel Assignment	Colorectal	III	N/R	Xiyuan Hospital of China Academy of Chinese Medical Sciences	NCT03716063
Fuzheng Yiliu Formula	Fuzheng Yiliu-1010	189 participants, Randomized, Parallel Assignment	Colorectal	II	Recruiting	Guangdong Provincial Hospital of Traditional Chinese Medicine	NCT04459754
Bushen-Jianpi Decoction	Study of TCM Syndrome of Hepatocellular Carcinoma and Colorectal Cancer Based on System Science	189 participants, Randomized, Single Group Assignment	Colorectal	I	N/R	Shanghai University of Traditional Chinese Medicine	NCT03189992
Liu-Jun-An-Wei Granule/ Qi-Tu-Er-Zhi Granule	Herbal Treatment to Improve Chemotherapy Delivery	400 participants, Randomized, Parallel Assignment	Colon	III	N/R	Xiyuan Hospital of China Academy of Chinese Medical Sciences	NCT03716518
Berberine hydrochloride	A Research of Berberine Hydrochloride to Prevent Colorectal Adenomas in Patients with Previous Colorectal Cancer	1000 participants, Randomized, Parallel Assignment	Colorectal	II-III	Completed	Xijing Hospital of Digestive Diseases	NCT03281096
Berberine hydrochloride	Study of Berberine Hydrochloride in Prevention of Colorectal Adenomas Recurrence	1108 participants, Randomized, Parallel Assignment	Colorectal	II-III	Completed	Shanghai Jiao Tong University School of Medicine	NCT02226185
Berberine hydrochloride	Primary Chemoprevention of Familial Adenomatous Polyposis with Berberine Hydrochloride	100 participants, Randomized, Parallel Assignment	Colorectal	II-III	Completed	Xijing Hospital of Digestive Diseases	NCT03333265
Berberine Chloride	Berberine Chloride in Preventing Colorectal Cancer in Patients with Ulcerative Colitis in Remission	18 participants, Randomized, Parallel Assignment	Colorectal	I	Completed	NCI	NCT02365480
Curcumin	Combining Curcumin with FOLFOX Chemotherapy in Patients with Inoperable Colorectal Cancer	41 participants, Randomized, Parallel Assignment	Colon	I-II	Completed	University of Leicester	NCT01490996
Curcumin	Curcumin for the Prevention of Colon Cancer	/	Colon	I	Completed	University of Michigan Rogel Cancer Center	NCT00027495
Curcumin C3 tablet	Curcumin Biomarkers	40 participants, Single Group Assignment	Colorectal	I	Completed	University of North Carolina, Chapel Hill	NCT01333917
Curcumin	Effect of Curcumin on Dose Limiting Toxicity and Pharmacokinetics of Irinotecan in Patients with Solid Tumors	23 participants, Non-Randomized, Single Group Assignment	Colorectal	I	Completed	UNC Lineberger Comprehensive Cancer Center	NCT01859858
Calcumin (Curcumin)	Use of Curcumin for Treatment of Intestinal Adenomas in Familial Adenomatous Polyposis (FAP)	44 participants, Randomized, Parallel Assignment	Intestinal	N/A	Completed	University of Puerto Rico	NCT00927485
Curcumin	Curcumin in Treating Patients with Familial Adenomatous Polyposis	44 participants, Randomized, Parallel Assignment	Intestinal	II	Completed	NCI	NCT00641147
Resveratrol	Resveratrol for Patients with Colon Cancer	11 participants, Single Group Assignment	Colon	I	Completed	University of California, Irvine	NCT00256334
Resveratrol	Resveratrol in Treating Patients with Colorectal Cancer That Can Be Removed by Surgery	20 participants, Non-Randomized, Single Group Assignment	Colorectal	I	Completed	NCI	NCT00433576
Artesunate	A Safety and Effectiveness Study of Pre-operative Artesunate in Stage II/​III Colorectal Cancer	200 participants, Randomized, Parallel Assignment	Colorectal	II	Recruiting	St George’s, University of London	NCT02633098
Pomegranate extract	Pomegranate Extract Supplementation in Colorectal Cancer Patients	60 participants, Randomized, Parallel Assignment	Colorectal	I-II	Completed	National Research Council, Spain	NCT01916239
Green tea extract	Green Tea Extracts for the Prevention of Colorectal Adenomas and Colorectal Cancer	176 participants, Randomized, Parallel Assignment	Colorectal	N/A	Completed	Seoul National University Hospital	NCT02321969
Green tea extract	Minimizing the Risk of Metachronous Adenomas of the Colorectum with Green Tea Extract	1001participants, Randomized, Parallel Assignment	Colorectal	II	Completed	Martin-Luther-Universität Halle-Wittenberg	NCT01360320
Annona muricata extract	Effect of Annona Muricata Leaves on Colorectal Cancer Patients and Colorectal Cancer Cells	30 participants, Randomized, Parallel Assignment	Colorectal	I	Completed	Indonesia University	NCT02439580
Ginger root extract	Ginger for Colorectal Cancer Prevention	30 participants, Randomized, Parallel Assignment	Colorectal	II	Completed	University of Michigan	NCT01344538
Ginger extract	Ginger and Gut Microbiome	68 participants, Randomized, Parallel Assignment	Colorectal	N/A	Completed	University of Minnesota	NCT03268655

The list above did not include those studies that were either suspended or terminated prematurely. (N/A, not applicable; N/R, not reported; NCI, National Cancer Institute.)

However, several challenges need to be addressed in translating CHM research into clinical practice. One major issue is the lack of standardization, as CHMs are often mixtures of herbs containing various bioactive compounds, making it difficult to ensure consistency in formulation and dosage across trials. This variability may hamper reproducibility and reliable efficacy assessment. Additionally, while preclinical studies have identified several bioactive compounds with anti-cancer properties, the specific molecular mechanisms of CHMs in the humans remain unclear, complicating the understanding of these multi-targeted therapies. Clinical trial design also poses challenges, as it must balance the individualized nature of CHMs with rigorous evidence-based standards. Finally, significant gaps remain in understanding the safety aspect of CHMs in humans, particularly when they are used for a longer duration [108]. More research will be needed to fully comprehend these aspects.

In summary, while CHM holds great potential, its benefits for IC are not yet been fully realized, warranting further investigation. Efforts should focus on standardization of CHMs, enhanced clinical trial designs, and comprehensive safety evaluations. Despite these challenges, ongoing trials provide a solid foundation for advancing CHM in personalized and evidence-based cancer care, offering valuable insights and creating new opportunities for improved IC treatment and patient care through the integrated Chinese and Western medicine (ICWM).

## Discussion and prospects

CHM has a rich development history and plays a significant role in preventing and treating malignant tumors. With its rich history and unique dialectical and holistic concepts, CM offers a different perspective on disease management compared to Western medicine. According to CM theory, illness arises from the imbalance of yin and yang, and the aim of CM is to restore this balance to alleviate the symptoms of the diseases [109, 110]. This holistic perspective not only targets the tumor directly, but also considers the overall well-being of the patients [111]. CM perceives cancer as a reflection of disrupted flow of Qi and blood and accumulation of phlegm turbidity in the body, and it emphasizes the importance of restoring the harmony of the bodily functions and the mental well-being for healing, which aligns well with modern medicine’s emphasis on addressing the patients’ mental and emotional health in cancer treatment [112]. The therapeutic effects of these herbs are linked to their abilities in terms of SBR, CHT, EDRP, and PBCRBS. Among these, herbs and CMF with the functions of SBR and CHT are the most frequently used in treatment of IC. The molecular mechanisms underlying the anti-IC effects are related to inhibition of cell proliferation, metastasis and angiogenesis, induction of apoptosis, reversal of chemoresistance and modulation of immune response. These herbs and their constituents, as well as CMF regulate many pathways to exert their anti-IC effects such as MAPK, NF-κB, PI3K/AKT and EMT.

As compared with the other chemotherapy drugs for IC treatment in Western medicine, CHMs have attracted great attention as potential therapeutic agents for cancer treatment in recent decades owing to their characteristics of multiple components, multi-targets and multi-pathways. CHMs are known to have several features such as displaying little toxicity and side effects and enhancing patient’s immunity [113], and improving quality of life during and after treatment [108, 114]. Moreover, the high cost of chemotherapy and target-therapy drugs prevent their wide acceptance in patients of developing countries, while relatively lower cost of CHMs could be a distinctive advantage for patients in many parts of the world. In contrast, Western medicine typically focuses on targeting the cancer cells directly through standardized treatments like surgery, chemotherapy, and radiation. Given the fundamental variance between Chinese and Western medicine approaches in treating disease, RCTs based on Western medicine diagnoses might not be the most suitable method for evaluating the effectiveness of CM. Additionally, there are some practical challenges in conducting clinical trials for CHMs, as CM does not adhere to the standardized “one-size-fits-all” approach of Western medicine; instead, CM formulae are often tailored for individual needs. Hence, using a pragmatic trial design could be better suited for evaluation of CHMs [115]. In this regard, CM philosophy seems to align with the contemporary concept of precision medicine used in oncology [116].

ICWM is a common approach of clinical practice in China. In ICWM, Western medicine provides targeted, evidence-based interventions, while CM contributes a holistic perspective that addresses the overall well-being of the patient. Western medicines might effectively reduce tumor size or eliminate cancer cells which could quickly manage the major symptoms of the patients with IC, but may cause severe side effects or drug resistance after long-term use. It has been reported that berberine, mainly derived from *Coptidis Rhizoma* and *Phellodendri Chinensis Cortex*, not only enhanced the tumor inhibitory effect of 5-FU in colorectal cancer [117], but also improved the 5-FU-induced intestinal mucosal injury by modulating the gut microbiota [118, 119]. In addition, curcumin, the main polyphenol isolated from *Curcumae Longae Rhizoma*, enhanced the tumor growth effect of 5-FU, oxaliplatin and bevacizumab in vitro and in vivo models of colorectal cancer, as well as reduced the side effects of bevacizumab [120–122]. Therefore, treatment of IC with ICWM offers a complementary approach that leverages the strengths of both systems [123]. However, to fully realize the potential of this integration, more investigations are warranted to confirm the efficacy and safety of ICWM for IC.

Despite the advantages of CHM in IC intervention, particularly CMF that often offer superior efficacy or lower toxicity compared to single herbs, there are still significant challenges to overcome before these natural products can be widely used in clinical application. As highlighted in this review, many natural products and CM formulations have shown promising anticancer activities against IC through multi-target mechanisms. However, the exact ingredients of CHMs have not yet been identified, and the exact action mechanisms of CMF are still unclear. Therefore, the path from promising natural compounds to clinically approved treatment modality remains long. Current clinical trials often fall short in providing robust theoretical support, and the specific mechanisms and potential adverse effects of some CHMs remain unclear, necessitating rigorous scientific investigation, including RCTs, to validate their efficacy and safety. Additionally, the intricate nature of CM requires extensive basic and clinical research for effective IC treatment. The inherent variability in CM treatment makes standardizing symptomatology and uniformly evaluating efficacy particularly challenging. Therefore, extensive research and rational standardization are crucial for the successful clinical application and promotion of CHM. Despite the many obstacles in developing natural compounds into anticancer drugs, there is a growing global effort to explore these possibilities in both preclinical and clinical settings. The search for new anti-IC agents from natural products remains a challenging yet exciting endeavor.

CHM offers a distinctive and comprehensive approach to manage IC, potentially enhancing patient outcomes and quality life. However, most chemo-preventative effects of these herbs have been studied in various human cancer cell lines, and to a lesser extent, in animal tumor models. Challenges such as the standardization of CHM formulations and rigorous clinical trial designs persist. Further research is essential to evaluate the therapeutic effects of CHMs for IC. More clinical trials and cohort studies are needed to establish the therapeutic benefits of these herbs.

## Electronic supplementary material

Below is the link to the electronic supplementary material.

Supplementary Material 1

## Data Availability

No datasets were generated or analysed during the current study.

## Abbreviations

5-FU: 5-Fluorouracil
CC: Colon cancer
CHM: Chinese herbal medicine
CHT: Clearing heat and toxin
CM: Chinese medicine
CMF: Chinese medicine formulae
CRC: Colorectal cancer
EDRP: Eliminating dampness and resolving phlegm
EMT: Epithelial–mesenchymal transition
GM-CSF: Granulocyte-macrophage colony-stimulating factor
HMGB1: High-mobility group box
IC: Intestinal cancer
ICWM: Integrated Chinese and Western medicine
PBCRBS: Promoting blood circulation to remove blood stasis
PZH: Pien Tze Huang
RCTs: Randomized controlled trials
SBR: Strengthening body resistance
N/A: Not applicable
NCI: National Cancer Institute
N/R: Not reported
