# Supplementary material for: Chinese herbal medicine for the treatment of intestinal cancer: preclinical studies and potential clinical applications
Source: Mol Cancer. 2024 Oct 1;23:217. doi: 10.1186/s12943-024-02135-3 (PMC11443726; doi:10.1186/s12943-024-02135-3)
Supplement: Supplementary file 1 — Supplementary Material 1 [file 12943_2024_2135_MOESM1_ESM.docx]

**Table S1** List of 61 classic formulae utilized for IC with ingredients

|  | **CM formulae** | **Compounds/Constituents** |
| --- | --- | --- |
| 1 | Si Jun Zi Decoction | *Ginseng Radix et Rhizoma* (Renshen), *Atractylodis Macrocephalae Rhizoma* (Baizhu), *Poria* (Fuling), and *Glycyrrhizae Radix et Rhizoma* (Gancao) |
| 2 | Jian Pi Hua Yu Decoction | *Pseudostellariae Radix* (Taizishen), *Coicis Semen* (Yiyiren), *Atractylodis Macrocephalae Rhizoma* (Baizhu), *Astragali Radix* (Huangqi), *Salviae Miltiorrhizae Radix et Rhizoma* (Danshen), *Scutellariae Barbatae Herba* (Banzhilian), *Paridis Rhizoma* (Chonglou), and *Curcumae Rhizoma* (Ezhu) |
| 3 | Jian Pi Jie Du Decoction | *Astragali Radix* (Huangqi), *Panacis Quinquefolii Radix* (Xiyangshen), *Atractylodis Macrocephalae Rhizoma* (Baizhu), *Poria* (Fuling), *Coicis Semen* (Yiyiren), *Smilacis Chinae Rhizoma* (Baqia), *Hedyotidis Herba* (Baihuasheshecao), *Scutellariae Barbatae Herba* (Banzhilian), *Paridis Rhizoma* (Chonglou), *Actinidiae Chinensis Fructus* (Mihoutao), and *Glycyrrhizae Radix et Rhizoma* (Gancao) |
| 4 | Baizhu Huangqi Decoction (also named *Astragalus atractylodes* Decoction) | *Astragali Radix* (Huangqi), *Atractylodis Macrocephalae Rhizoma* (Baizhu), *Curcumae Rhizoma* (Ezhu), *Actinidiae Chinensis Radix* (Tengligen), *Benincasae Cortex* (Dongguapi), and *Fici Pumilae Caulis* (Bili) |
| 5 | Xiang Sha Liu Jun Zi Decoction | *Aucklandiae Radix* (Muxiang), *Amomi Fructus* (Sharen) , *Citri Reticulatae Pericarpium* (Chenpi), *Pinelliae Rhizoma* (Banxia), *Codonopsis Radix* (Dangshen), *Atractylodis Macrocephalae Rhizoma* (Baizhu), *Poria* (Fuling), *Glycyrrhizae Radix et Rhizoma* (Gancao), *Zingiberis Rhizoma* (Ganjiang), and *Jujubae Fructus* (Dazao) |
| 6 | Shen Ling Bai Zhu San | *Ginseng Radix et Rhizoma* (Renshen), *Atractylodis Macrocephalae Rhizoma* (Baizhu), *Poria* (Fuling), *Wikstroemiae Indicae Radix* (Liaogewang), *Ficus Simplicissimae Radix* (Wuzhualong), *Polygonum Chinense Herba* (Huotanmu), *Kalopanax Septemlobus Cortex* (Ciqiu), *Ailanthus altissima Swingle* (Chunpi), *Coicis Semen* (Yiyiren), and *Curcumae Rhizoma* (Ezhu) |
| 7 | Yi Qi Fu Sheng Formula | *Codonopsis Radix* (Dangshen), *Atractylodis Macrocephalae Rhizoma* (Baizhu), *Poria* (Fuling), *Glycyrrhizae Radix et Rhizoma* (Gancao), *Myristicae Semen* (Roudoukou) and *Akebia Fructus* (Bayuezha) |
| 8 | Ba Zhen Decoction | *Ginseng Radix et Rhizoma* (Renshen), *Atractylodis Macrocephalae Rhizoma* (Baizhu), *Poria* (Fuling), *Angelicae Sinensis Radix* (Danggui), *Chuanxiong Rhizoma* (Chuanxiong), *Paeoniae Radix Alba* (Baishao), *Rehmanniae Radix Praeparata* (Shudihuang), and *Glycyrrhizae Radix et Rhizoma* (Gancao) |
| 9 | Si Ni Decoction | *Aconiti Lateralis Radix Praeprarata* (Fuzi), *Glycyrrhizae Radix et Rhizoma* (Gancao), and *Zingiberis Rhizoma* (Ganjiang) |
| 10 | Bu Shen Jian Pi Jie Du Decoction | *Astragali Radix* (Huangqi), *Rehmanniae Radix Praeparata* (Shudihuang), *Atractylodis Macrocephalae Rhizoma* (Baizhu), *Corni Fructus* (Shanzhuyu), *Dioscoreae Rhizoma* (Shanyao), *Sophorae Flavescentis Radix* (Kushen), *Vitis quinquangularis Rehd Folium* (Maoputaoye), and *Akebiae Caulis* (Mutong) |
| 11 | Fu Zheng Xiao Ai Decoction | *Astragali Radix* (Huangqi), *Atractylodis Macrocephalae Rhizoma* (Baizhu), *Poria* (Fuling), *Dioscoreae Rhizoma* (Shanyao), *Pseudostellariae Radix* (Taizishen), *Hedyotidis Herba* (Baihuasheshecao), *Cremastrae Pseudobulbus Pleiones Pseudobulbus* (Shancigu), *Chinese Actinidia Radix* (Tengligen), *Glycyrrhizae Radix et Rhizoma* (Gancao) |
| 12 | Dang Gui Bu Xue Decoction | *Astragali Radix* (Huangqi), and *Angelicae Sinensis Radix* (Danggui) |
| 13 | Pien Tze Huang | *Bovis Calculus* (Niuhuang), *Fel*Seprentis (Shedan), and *Notoginseng Radix et Rhizoma* (Sanqi) |
| 14 | Wei Tong Xin | *Rhei Radix et Rhizoma* (Dahuang), *Pharbitidis Semen* (Qianniuzi), *Auchlandiae Radix* (Muxiang), *Gleditsia Sinensis* (Zaojia), and *Glycyrrhizae Radix et Rhizoma* (Gancao) |
| 15 | Ban Xia Xie Xin Decoction | *Pinelliae Rhizoma* (Banxia), *Scutellariae Radix* (Huangqin), *Zingiberis Rhizoma* (Ganjiang), *Ginseng Radix et Rhizoma* (Renshen), *Glycyrrhizae Radix et Rhizoma* (Gancao), *Coptidis Rhizoma* (Huanglian), and *Jujubae Fructus* (Dazao) |
| 16 | Xian Lian Jie Du Decoction | *Agrimoniae Herba* (Xianhecao), *Coptidis Rhizoma* (Huanglian), *Sophorae Flavescentis Radix* (Kushen), *Coicis Semen* (Yiyiren), *Sparganii Rhizoma* (Sanleng), *Curcumae Rhizoma* (Ezhu), *Astragali Radix* (Huangqi), and *Atractylodis Macrocephalae Rhizoma* (Baizhu) |
| 17 | Huang Lian Jie Du Decoction | *Coptidis Rhizoma* (Huanglian), *Phellodendri Amurensis Cortex* (Guanhuangbo), *Gardeniae Fructus* (Zhizi), and *Scutellariae Radix* (Huangqin) |
| 18 | Jie Du San Gen Decoction | *Chinese Actinidia Radix* (Tengligen*), Geum Aleppicum Radix* (Shuiyangmeigen), and *Polygoni Cuspidati Radix* (Huzhanggen) |
| 19 | Xiao Ai Jie Du Decoction | *Hedyotidis Herba* (Baihuasheshecao), *Scutellariae Barbatae Herba* (Banzhilian), *Pseudostellariae Radix* (Taizishen), *Ophiopogonis Radix* (Maidong), *Cremastrae Pseudobulbus Pleiones Pseudobulbus* (Shancigu), *Curcumae Rhizoma* (Ezhu), and *Akebiae Fructus* (Yuzhizi) |
| 20 | Huang Qin Decoction | *Scutellariae Radix* (Huangqin), *Paeoniae Radix Alba* (Baishao), *Jujubae Fructus* (Dazao), and *Glycyrrhizae Radix et Rhizoma* (Gancao) |
| 21 | San Wu Huang Qin Decoction | *Scutellariae Radix* (Huangqin), *Sophorae Flavescentis Radix* (Kushen), and *Rehmanniae Radix Praeparata* (Shudihuang) |
| 22 | Ge Gen Qin Lian Decoction | *Puerariae Lobatae Radix* (Gegen), *Scutellariae Radix* (Huangqin), *Coptidis Rhizoma* (Huanglian), and *Glycyrrhizae Radix et Rhizoma* (Gancao) |
| 23 | Da Cheng Qi Decoction | *Rhei Radix et Rhizoma* (Dahuang), *Magnoliae Officibalis Cortex* (Houpo), *Aurantii Fructus Immaturus* (Zhishi), and *Natrii Sulfas* (Mangxiao) |
| 24 | Teng Long Bu Zhong Decoction | *Chinese Actinidia Radix* (Tengligen), *Solani Nigri Herba* (Longkui), *Duchesnea Indica Fructus* (Shemei), *Atractylodis Macrocephalae Rhizoma* (Baizhu), *Poria* (Fuling), *Coicis Semen* (Yiyiren), *Visci Herba* (Hujisheng), and *Scutellariae Barbatae Herba* (Banzhilian) |
| 25 | Zuo Jin Wan | *Coptidis Rhizoma* (Huanglian), and *Euodiae Fructus* (Wuzhuyu) |
| 26 | Er Chen Decoction | *Pinelliae Rhizoma* (Banxia), *Citri Reticulatae Pericarpium* (Chenpi), *Poria* (Fuling), and *Glycyrrhizae Radix et Rhizoma* (Gancao) |
| 27 | Shao Yao Decoction | *Paeoniae Radix Alba* (Baishao), *Angelicae Sinensis Radix* (Danggui), *Coptidis Rhizoma* (Huanglian), *Arecae Semen* (Binlang), *Aucklandiae Radix* (Muxiang), *Glycyrrhizae Radix et Rhizoma* (Gancao), *Rhei Radix et Rhizoma* (Dahuang), *Scutellariae Radix* (Huangqin), and *Cinnamomi Cortex* (Rougui) |
| 28 | San Jie Yi Liu Formula | *Pinelliae Rhizoma Preparatum* (Fabanxia), *Sarcandrae Herba* (Zhongjiefeng), *Fritillariae Thunbergii Bulbus* (Zhebeimu), and *Eupolyphage Steleophaga* (Tubiechong) |
| 29 | Jian Pi Jie Du Formula | *Astragali Radix* (Huangqi), *Atractylodis Macrocephalae Rhizoma* (Baizhu), *Vitis Quinquangularis Caulis* (Yeputaoteng), *Akebia Fructus* (Bayuezha), *Salviae Chinensia Herba* (Shijianchuan), and *Euodiae Fructus* (Wuzhuyu) |
| 30 | Fu Fang Chang Wei Qing Formula | *Astragali Radix* (Huangqi), *Atractylodis Macrocephalae Rhizoma* (Baizhu), *Codonopsis Radix* (Dangshen), *Polyporus* (Zhuling), *Akebia Fructus* (Bayuezha), *Coicis Semen* (Yiyiren), *Vitis Quinquangularis Caulis* (Yeputaoteng), and *Sargentodoxa cuneata* (Hongteng) |
| 31 | Jian Pi Xiao Ai Formula | *Ginseng Radix et Rhizoma* (Renshen), *Coicis Semen* (Yiyiren), *Epimedii Folium* (Yinyanghuo), *Aurantii Fructus* (Zhike), *Hedyotidis Herba* (Baihuasheshecao), *Paridis Rhizoma* (Chonglou), *Chinese Actinidia Radix* (Tengligen), and *Curcumae Radix* (Yujin) |
| 32 | Jiang Pi Qing Re Huo Xue Formula | *Ilicis Rotundae Cortex* (Jiubiying), *Hirudo* (Shuizhi), *Notoginseng Radix et Rhizoma* (Sanqi), *Atractylodis Macrocephalae Rhizoma* (Baizhu), *Paeoniae Radix Alba* (Baishao), *Glycyrrhizae Radix et Rhizoma* (Gancao) |
| 33 | Fu Zheng Jian Pi Formula | *Codonopsis Radix* (Dangshen), *Poria* (Fuling), and *Akebia Fructus* (Bayuezha) |
| 34 | Bu Shen Jian Pi Formula | *Astragali Radix* (Huangqi), *Atractylodis Macrocephalae Rhizoma* (Baizhu), *Ligustri lucidi Fructus* (Nvzhenzi), *Cistanches Herba* (Roucongrong), *Vitis Quinquangularis Caulis* (Yeputaoteng), *Salviae Chinensia Herba* (Shijianchuan), and *Akebia Fructus* (Bayuezha) |
| 35 | Jian Pi Yi Shen Formula | *Astragali Radix* (Huangqi), *Atractylodis Macrocephalae Rhizoma* (Baizhu), *Eucommiae Cortex* (Duzhong), *Epimedii Folium* (Yinyanghuo), *Lycii Fructus* (Gouqizi), *Dioscoreae Rhizoma* (Shanyao), *Codonopsis Radix* (Dangshen), and *Poria* (Fuling) |
| 36 | Jian Pi Shen Shi Formula | *Codonopsis Radix* (Dangshen), *Poria* (Fuling), *Citri Reticulatae Pericarpium* (Chenpi), *Atractylodis Macrocephalae Rhizoma* (Baizhu), *Pinelliae Rhizoma* (Banxia), *Aurantii Fructus Immaturus* (Zhishi), *Magnoliae Officibalis Cortex* (Houpo), *Coicis Semen* (Yiyiren), *Hedyotidis Herba* (Baihuasheshecao), *Scutellariae Barbatae Herba* (Banzhilian), and *Glycyrrhizae Radix et Rhizoma* (Gancao) |
| 37 | Yi Qi Jian Pi Hua Shi Formula | *Codonopsis Radix* (Dangshen), *Astragali Radix* (Huangqi), *Atractylodis Macrocephalae Rhizoma* (Baizhu), *Salviae Chinensia Herba* (Shijianchuan), *Hedyotidis Herba* (Baihuasheshecao), *Curcumae Rhizoma* (Ezhu), *Sparganii Rhizoma* (Sanleng), *Paeoniae Radix Alba* (Baishao), *Pinelliae Rhizoma* (Banxia), *Citri Reticulatae Pericarpium* (Chenpi), *Angelicae Sinensis Radix* (Danggui), and *Glycyrrhizae Radix et Rhizoma* (Gancao) |
| 38 | Qing Re Hua Shi Jian Pi Xiao Zheng Formula | *Angelicae Sinensis Radix* (Danggui), *Dioscoreae Rhizoma* (Shanyao), *Atractylodis Macrocephalae Rhizoma* (Baizhu), *Pinelliae Rhizoma* (Banxia), *Coicis Semen* (Yiyiren), *Curcumae Rhizoma* (Ezhu), *Sophorae Flavescentis Radix* (Kushen), *Fritillariae Thunbergii Bulbus* (Zhebeimu), *Patriniae Herba* (Baijiangcao), *Codonopsis Radix* (Dangshen), *Poria* (Fuling), *Solani Nigri Herba* (Longkui), *Glycyrrhizae Radix et Rhizoma* (Gancao), and *Citri Reticulatae Pericarpium* (Chenpi) |
| 39 | Qing Jie Fu Zheng Particle | *Hedyotidis Herba* (Baihuasheshecao), *Scutellariae Barbatae Herba* (Banzhilian), *Hordei Fructus Germinatus* (Maiya), and *Astragali Radix* (Huangqi) |
| 40 | Yi Qi Jie Du Formula | *Codonopsis Radix* (Dangshen), *Astragali Radix* (Huangqi), *Coptidis Rhizoma* (Huanglian), *Hedyotidis Herba* (Baihuasheshecao), *Trichosanthis Radix* (Tianhuafen), *Poria* (Fuling), and *Glycyrrhizae Radix et Rhizoma* (Gancao) |
| 41 | Yi Yuan Decoction | *Astragali Radix* (Huangqi), *Ginseng Radix et Rhizoma* (Shengshaishen), *Polyporus* (Zhuling), *Poria* (Fuling), *Lycii Fructus* (Gouqizi), and *Ligustri lucidi Fructus* (Nvzhenzi) |
| 42 | Ge Gen Decoction | *Cinnamomi Cortex* (Rougui), *Glycyrrhizae Radix et Rhizoma* (Gancao), *Paeoniae Alba Radix* (Shaoyao), *Zingiberis Rhizoma* (Ganjiang), and *Jujubae Fructus* (Dazao) |
| 43 | Fu Zheng Xiao Ji Formula | *Astragali Radix* (Huangqi), *Atractylodis Macrocephalae Rhizoma* (Baizhu), *Ganoderma* (Lingzhi), *Coicis Semen* (Yiyiren), *Hedyotidis Herba* (Baihuasheshecao), *Scorpio* (Quanxie), *Prunellae Spica* (Xiakucao), and *Curcumae Rhizoma* (Ezhu) |
| 44 | Qi Zhen Capsule | *Astragali Radix* (Huangqi), *Ginseng Radix et Rhizoma* (Renshen), *Isatidis Folium* (Daqingye), *Paridis Rhizoma* (Chonglou), and *Margarita* (Zhenzhu) |
| 45 | Chang Fu Formula | *Astragali Radix* (Huangqi), *Atractylodis Macrocephalae Rhizoma* (Baizhu), *Citri Reticulatae Pericarpium* (Chenpi), *Hordei Fructus Germinatus* (Maiya), *Galli Gigerii Endothelium Corneum* (Jineijin), *Coicis Semen* (Yiyiren), *Cyperi Rhizoma* (Xiangfu), *Curcumae Radix* (Yujin), *Curcumae Rhizoma* (Ezhu), *Eupolyphage Steleophaga* (Tubiechong), *Scutellariae Barbatae Herba* (Banzhilian), *Hedyotidis Herba* (Baihuasheshecao), *Gecko* (Bihu), and *Glycyrrhizae Radix et Rhizoma* (Gancao) |
| 46 | Chang Fu Kang | *Brucea Fructus* (Yadanzi), *Camptotheca acuminata* Decne. (Xishuguo), *Ginseng Radix et Rhizoma* (Renshen), and *Curcumae Rhizoma* (Ezhu) |
| 47 | Fu Fang Chang Tai Formula | *Ginseng Radix et Rhizoma* (Renshen), *Coicis Semen* (Yiyiren), *Curcumae Rhizoma* (Ezhu), *Akebia Fructus* (Bayuezha), *Hedyotidis Herba* (Baihuasheshecao), *Chinese Actinidia Radix* (Tengligen), and *Geotrupidae* (Shikelang) |
| 48 | Zang Du Qing | *Angelicae Sinensis Radix* (Danggui), *Codonopsis Radix* (Dangshen), *Atractylodis Macrocephalae Rhizoma* (Baizhu), *Scutellariae Barbatae Herba* (Banzhilian), *Cremastrae Pseudobulbus Pleiones Pseudobulbus* (Shancigu), *Hedyotidis Herba* (Baihuasheshecao), *Pulsatillae Radix* (Baitouweng), and *Trionycis Carapax Praeparata Cum Melle* (Zhibiejia) |
| 49 | Fu Zheng Yi Ai Formula | *Codonopsis Radix* (Dangshen), *Astragali Radix* (Huangqi), *Salviae Chinensia Herba* (Shijianchuan), *Atractylodis Macrocephalae Rhizoma* (Baizhu), and *Hedyotidis Herba* (Baihuasheshecao) |
| 50 | E Zhu Kang Ai Formula | *Astragali Radix* (Huangqi), *Curcumae Rhizoma* (Ezhu), *Paridis Rhizoma* (Chonglou), *Agrimoniae Herba* (Xianhecao), *Asparagi Radix* (Tiandong), and *Pinelliae Rhizoma* (Banxia) |
| 51 | Yi Ai Formula | *Astragali Radix* (Huangqi), *Curcumae Rhizoma* (Ezhu), *Ficus Pumilae* *Fructus* (Mumantou), *Chinese Actinidia Radix* (Tengligen), and *Benincasae Semen* (Dongguazi) |
| 52 | Bu Shen Jie Du San Jie Formula | *Rehmanniae Radix* (Dihuang), *Ligustri lucidi Fructus* (Nvzhenzi), *Cistanches Herba* (Roucongrong), *Cremastrae Pseudobulbus Pleiones Pseudobulbus* (Shancigu), and *Akebia Fructus* (Bayuezha) |
| 53 | Bu Zhong Gu Ben Yi Wei Formula | *Atractylodis Macrocephalae Rhizoma* (Baizhu), *Astragali Radix* (Huangqi), *Ginseng Radix et Rhizoma* (Renshen), *Poria* (Fuling), *Citri Reticulatae Pericarpium* (Chenpi), *Patriniae Herba* (Baijiangcao), *Cremastrae Pseudobulbus Pleiones Pseudobulbus* (Shancigu), *Angelicae Sinensis Radix* (Danggui), *Chuanxiong Rhizoma* (Chuanxiong), *Paeoniae Radix Alba* (Baishao), *Bupleuri Radix* (Chaihu), *Amomi Fructus* (Sharen), *Glycyrrhizae Radix et Rhizoma* (Gancao), *Zingiberis Rhizoma Recens* (Shengjiang), and *Jujubae Fructus* (Dazao) |
| 54 | Fu Fang Shen Qi Decoction | *Panax notoginseng* (Shensanqi), *Astragali Radix* (Huangqi), *Ginseng Radix et Rhizoma* (Renshen), *Angelicae Sinensis Radix* (Danggui), *Citri Reticulatae Pericarpium* (Chenpi), *Aucklandiae Radix* (Muxiang), *Aconiti Radix* (Chuancaowu), *Scutellariae Barbatae Herba* (Banzhilian), *Lobeliae Chinensis Herba* (Banbianlian), *Glycyrrhizae Radix et Rhizoma* (Gancao), and *Fructus Setariae Germinatus* (Gumaiya) |
| 55 | Fu Zheng Mixture | *Schisandrae Chinensis Fructus* (Wuweizi), *Aloe* (Luhui), and *Astragali Radix* (Huangqi) |
| 56 | E Huang Decoction | *Curcumae Rhizoma* (Ezhu), *Coicis Semen* (Yiyiren), *Laminariae Thallus Eckloniae Thallus* (Kunbu), and *Rhei Radix et Rhizoma* (Dahuang) |
| 57 | Huo Xue Hua Yu JC734 | *Ginseng Radix et Rhizoma* (Renshen), *Cinnamomi Cortex* (Rougui), *Sparganii Rhizoma* (Sanleng), *Curcumae Rhizoma* (Ezhu), and *Hirudo* (Shuizhi) |
| 58 | Gui Zhi Fu Ling Pill | *Cinnamomi Ramulus* (Guizhi), *Poria* (Fuling), *Paeoniae Alba* *Radix* (Shaoyao), *Moutan Cortex* (Danpi), and *Persicae Semen* (Taoren) |
| 59 | Di Dang Decoction | *Tabanus* (Mangchong), *Hirudo* (Shuizhi), *Persicae Semen* (Taoren), *Rhei Radix et Rhizoma* (Dahuang) |
| 60 | Dan Shen Yin | *Salviae Miltiorrhizae Radix et Rhizoma* (Danshen), *Santali Albilignum* (Tanxiang), *Amomi Fructus* (Sharen) |
| 61 | Yang Yin Hua Yu Jie Du Formula | *Paeoniae Radix Alba* (Baishao), *Patriniae Herba* (Baijiangcao), *Poria* (Fuling), *Codonopsis Radix* (Dangshen), *Scutellariae Radix* (Huangqin), *Coptidis Rhizoma* (Huanglian), *Angelicae Sinensis Radix* (Danggui), *Atractylodis Macrocephalae Rhizoma* (Baizhu), *Saposhnikoviae Radix* (Fangfeng), *Aucklandiae Radix* (Muxiang), *Rhei Radix et Rhizoma* (Dahuang), *Notoginseng Radix et Rhizoma* (Sanqi), *Cinnamomi Cortex* (Rougui), and *Glycyrrhizae Radix et Rhizoma* (Gancao) |

**Table S2** List of 119 herbs and frequency in IC

| **Herb name in Chinese** | **Pharmaceutical name** | **Frequency of occurrence** | **Frequency ratio (n=69)** |
| --- | --- | --- | --- |
| Baizhu | *Atractylodis Macrocephalae Rhizoma* | 26 | 37.68% |
| Huangqi | *Astragali Radix* | 24 | 34.78% |
| Gancao | *Glycyrrhizae Radix et Rhizoma* | 23 | 33.33% |
| Fuling | *Poria* | 18 | 26.09% |
| Ezhu | *Curcumae Rhizoma* | 15 | 21.74% |
| Yiyiren | *Coicis Semen* | 13 | 18.84% |
| Baihuasheshecao | *Hedyotidis Herba* | 13 | 18.84% |
| Dangshen | *Codonopsis Radix* | 12 | 17.39% |
| Renshen | *Ginseng Radix et Rhizoma* | 11 | 15.94% |
| Banzhilian | *Scutellariae Barbatae Herba* | 9 | 13.04% |
| Danggui | *Angelicae Sinensis Radix* | 9 | 13.04% |
| Huanglian | *Coptidis Rhizoma* | 8 | 11.59% |
| Banxia | *Pinelliae Rhizoma* | 8 | 11.59% |
| Chenpi | *Citri Reticulatae Pericarpium* | 8 | 11.59% |
| Huangqin | *Scutellariae Radix* | 7 | 10.14% |
| Baishao | *Paeoniae Radix Alba* | 7 | 10.14% |
| Tengligen | *Actinidia Chinese Radix* | 7 | 10.14% |
| Bayuezha | *Akebia Fructus* | 7 | 10.14% |
| Dahuang | *Rhei Radix et Rhizoma* | 6 | 8.70% |
| Chonglou | *Paridis Rhizoma* | 5 | 7.25% |
| Shancigu | *Cremastrae Pseudobulbus Pleiones Pseudobulbus* | 5 | 7.25% |
| Muxiang | *Auchlandiae Radix* | 5 | 7.25% |
| Dazao | *Jujubae Fructus* | 4 | 5.80% |
| Rougui | *Cinnamomi Cortex* | 4 | 5.80% |
| Kushen | *Sophorae Flavescentis Radix* | 4 | 5.80% |
| Shijianchuan | *Salviae Chinensia Herba* | 4 | 5.80% |
| Shanyao | *Dioscoreae Rhizoma* | 4 | 5.80% |
| Baijiangcao | *Patriniae Herba* | 3 | 4.35% |
| Taizishen | *Pseudostellariae Radix* | 3 | 4.35% |
| Nuzhenzi | *Ligustri lucidi Fructus* | 3 | 4.35% |
| Shudihuang | *Rehmanniae Radix Praeparata* | 3 | 4.35% |
| Sharen | *Amomi Fructus* | 3 | 4.35% |
| Ganjiang | *Zingiberis Rhizoma* | 3 | 4.35% |
| Yeputaoteng | *Vitis Quinquangularis Caulis* | 3 | 4.35% |
| Sanleng | *Sparganii Rhizoma* | 3 | 4.35% |
| Shuizhi | *Hirudo* | 3 | 4.35% |
| Sanqi | *Notoginseng Radix et Rhizoma* | 3 | 4.35% |
| Yujin | *Curcumae Radix* | 2 | 2.90% |
| Zhishi | *Aurantii Fructus Immaturus* | 2 | 2.90% |
| Roucongrong | *Cistanches Herba* | 2 | 2.90% |
| Zhebeimu | *Fritillariae Thunbergii Bulbus* | 2 | 2.90% |
| Taoren | *Persicae Semen* | 2 | 2.90% |
| Xianhecao | *Agrimoniae Herba* | 2 | 2.90% |
| Gouqizi | *Lycii Fructus* | 2 | 2.90% |
| Shaoyao | *Paeoniae Alba Radix* | 2 | 2.90% |
| Longkui | *Solani Nigri Herba* | 2 | 2.90% |
| Dongguapi | *Benincasae Cortex* | 2 | 2.90% |
| Dongguazi | *Benincasae Semen* |  |  |
| Danshen | *Salviae Miltiorrhizae Radix et Rhizoma* | 2 | 2.90% |
| Chuanxiong | *Chuanxiong Rhizoma* | 2 | 2.90% |
| Zhuling | *Polyporus* | 2 | 2.90% |
| Houpo | *Magnoliae Officibalis Cortex* | 2 | 2.90% |
| Yinyanghuo | *Epimedii Folium* | 2 | 2.90% |
| Maiya | *Hordei Fructus Germinatus* | 2 | 2.90% |
| Wuzhuyu | *Euodiae Fructus* | 2 | 2.90% |
| Tubiechong | *Eupolyphage Steleophaga* | 2 | 2.90% |
| Yadanzi | *Brucea Fructus* | 1 | 1.45% |
| Baitouweng | *Pulsatillae Radix* | 1 | 1.45% |
| Daqingye | *Isatidis Folium* | 1 | 1.45% |
| Jineijin | *Galli Gigerii Endothelium Corneum* | 1 | 1.45% |
| Shedan | *Fel*Seprentis | 1 | 1.45% |
| Maidong | *Ophiopogonis Radix* | 1 | 1.45% |
| Baqia | *Smilacis Chinae Rhizoma* | 1 | 1.45% |
| Ciqiu | *Kalopanax Septemlobus Cortex* | 1 | 1.45% |
| Tanxiang | *Santali Albilignum* | 1 | 1.45% |
| Xiangfu | *Cyperi Rhizoma* | 1 | 1.45% |
| Chuancaowu | *Aconiti Radix* | 1 | 1.45% |
| Shanzhuyu | *Corni Fructus* | 1 | 1.45% |
| Wuzhualong | *Ficus Simplicissimae Radix* | 1 | 1.45% |
| Mangxiao | *Natrii Sulfas* | 1 | 1.45% |
| Zhizi | *Gardeniae Fructus* | 1 | 1.45% |
| Hongteng | *Sargentodoxae Caulis* | 1 | 1.45% |
| Binlang | *Arecae Semen* | 1 | 1.45% |
| Xiyangshen | *Panacis Quinquefolii Radix* | 1 | 1.45% |
| Wuweizi | *Schisandrae Chinensis Fructus* | 1 | 1.45% |
| Shengshaishen | *Ginseng Radix et Rhizoma* | 1 | 1.45% |
| Fuzi | *Aconiti Lateralis Radix Praeprarata* | 1 | 1.45% |
| Shengjiang | *Zingiberis Rhizoma Recens* | 1 | 1.45% |
| Lingzhi | *Ganoderma* | 1 | 1.45% |
| Maoputaoye | *Vitis quinquangularis Rehd Folium* | 1 | 1.45% |
| Chaihu | *Bupleuri Radix* | 1 | 1.45% |
| Huotanmu | *Polygonum chinense Herba* | 1 | 1.45% |
| Guanhuangbo | *Phellodendri Amurensis Cortex* | 1 | 1.45% |
| Roudoukou | *Myristicae Semen* | 1 | 1.45% |
| Shensanqi | *Panax notoginseng* | 1 | 1.45% |
| Xishuguo | *Camptotheca acuminata* Decne. | 1 | 1.45% |
| Niuhuang | *Bovis Calculus* | 1 | 1.45% |
| Liaogewang | *Lablab Wikstroemia indica* | 1 | 1.45% |
| Luhui | *Aloe* | 1 | 1.45% |
| Zaojia | *Gleditsia Sinensis* | 1 | 1.45% |
| Huzhanggen | *Polygoni Cuspidati Radix* | 1 | 1.45% |
| Shikelang | *Geotrupidae* | 1 | 1.45% |
| Tiandong | *Asparagi Radix* | 1 | 1.45% |
| Zhenzhu | *Margarita* | 1 | 1.45% |
| Shuiyangmeigen | *Geum Aleppicum Radix* | 1 | 1.45% |
| Bihu | *Gecko* | 1 | 1.45% |
| Hujisheng | *Visci Herba* | 1 | 1.45% |
| Guizhi | *Cinnamomi Ramulus* | 1 | 1.45% |
| Gumaiya | *Fructus Setariae Germinatus* | 1 | 1.45% |
| Shemei | *Duchesnea Indicae Fructus* | 1 | 1.45% |
| Zhongjiefeng | *Sarcandrae Herba* | 1 | 1.45% |
| Duzhong | *Eucommiae Cortex* | 1 | 1.45% |
| Gegen | *Puerariae Lobatae Radix* | 1 | 1.45% |
| Fangfeng | *Saposhnikoviae Radix* | 1 | 1.45% |
| Mihoutao | *Actinidia Chinese Fructus* | 1 | 1.45% |
| Quanxie | *Scorpio* | 1 | 1.45% |
| Zhibiejia | *Trionycis Carapax Praeparata Cum Melle* | 1 | 1.45% |
| Banbianlian | *Lobeliae Chinensis Herba* | 1 | 1.45% |
| Tianhuafen | *Trichosanthis Radix* | 1 | 1.45% |
| Xiakucao | *Prunellae Spica* |  | 1.45% |
| Mutong | *Akebiae Caulis* | 1 | 1.45% |
| Danpi | *Moutan Cortex* | 1 | 1.45% |
| Mangchong | *Tabanus* | 1 | 1.45% |
| Zhike | *Aurantii Fructus* | 1 | 1.45% |
| Dihuang | *Rehmanniae Radix* | 1 | 1.45% |
| Bili | *Fici Pumilae Caulis* | 1 | 1.45% |
| Yuzhizi | *Akebiae Fructus* | 1 | 1.45% |
| Qianniuzi | *Pharbitidis Semen* | 1 | 1.45% |
| Mumantou | *Ficus Pumilae Fructus* | 1 | 1.45% |
| Kunbu | *Laminariae Thallus Eckloniae Thallus* | 1 | 1.45% |
| Jiubiying | *Ilicis Rotundae Cortex* | 1 | 1.45% |
| Chunpi | *Ailanthi Cortex* | 1 | 1.45% |
| **Total** |  | **398** |  |
